# ﻿*Desmopsisterriflora*, an extraordinary new species of Annonaceae with flagelliflory

**DOI:** 10.3897/phytokeys.227.102279

**Published:** 2023-06-23

**Authors:** María Fernanda Martínez-Velarde, Carlos Rodrigues-Vaz, Vincent Soulé, Francis J. Nge, George E. Schatz, Thomas L. P. Couvreur, Andrés Ernesto Ortiz-Rodriguez

**Affiliations:** 1 Posgrado en Ciencias biológicas, Universidad Nacional Autónoma de México (UNAM), Ciudad de México, Mexico; 2 Departamento de Botánica, Instituto de Biología, Universidad Nacional Autónoma de México (UNAM), Ciudad de México, Mexico; 3 Institut de Systématique, Evolution, Biodiversité (ISYEB), Muséum National d‘Histoire Naturelle-CNRS-SU-EPHE-UA, Paris, France; 4 DIADE, Université de Montpellier, CIRAD, IRD, Montpellier, France; 5 Missouri Botanical Garden, P.O. Box 299, St. Louis, MO 63166-0299, USA

**Keywords:** Anatomy, Cauliflory, Mexico, phylogeny, tropical rain forest

## Abstract

Flagelliflory refers to the production of inflorescences exclusively on long, whip-like branches which emerge from the main trunk and extend along the ground or below it. It is the rarest type of cauliflory and only a few cases have been reported in the world. Here, a new species of Annonaceae with flagelliflory is described and illustrated. The phylogenetic relationships of the new species were inferred using a hybrid-capture phylogenomic approach and we present some notes on its reproductive ecology and pollen characteristics. The new species, namely *Desmopsisterriflora***sp. nov.**, is part of a clade composed of Mexican species of *Stenanona* with long, awned petals. *Desmopsisterriflora* is distinguished by its flageliflorous inflorescences, basely fused sepals, thick red petals, reduced number of ovules per carpel, pollen grains with a weakly rugulate to fossulate exine ornamentation, and its globose, apiculate fruits with a woody testa. The morphological characteristics of the flagella suggest that these are specialized branches rather than inflorescences, and the absence of ramiflory implies an exclusively reproductive function. The flowers are infrequently visited by insects, their potential pollinators being flies and ants.

## ﻿Introduction

Cauliflory is the general term used to refer to the production of inflorescences along the main trunk and on leafless branches of woody plants (Richards 1996). It is a common phenomenon in tropical forests and rarely seen in temperate ecosystems. In very dense forests, cauliflory facilitates the visit of plant pollinators and seed dispersers, since its flowers and fruits are more visible and easily accessible (Richards 1996). Flagelliflory can be seen as an extreme and spectacular case of cauliflory, referring to the production of inflorescences exclusively on long, whip-like branches (flagella), sometimes with scale leaves and long internodes. The flagella arise from the main trunk and then extend along the surface of the ground or slightly below it and most of the flowers appear to rise directly from the soil (Milbraed 1922, Fig. 1). Thus, flagelliflory has a strictly reproductive function since the flowers only grow on the flagella and not on branches.

**Figure 1.**
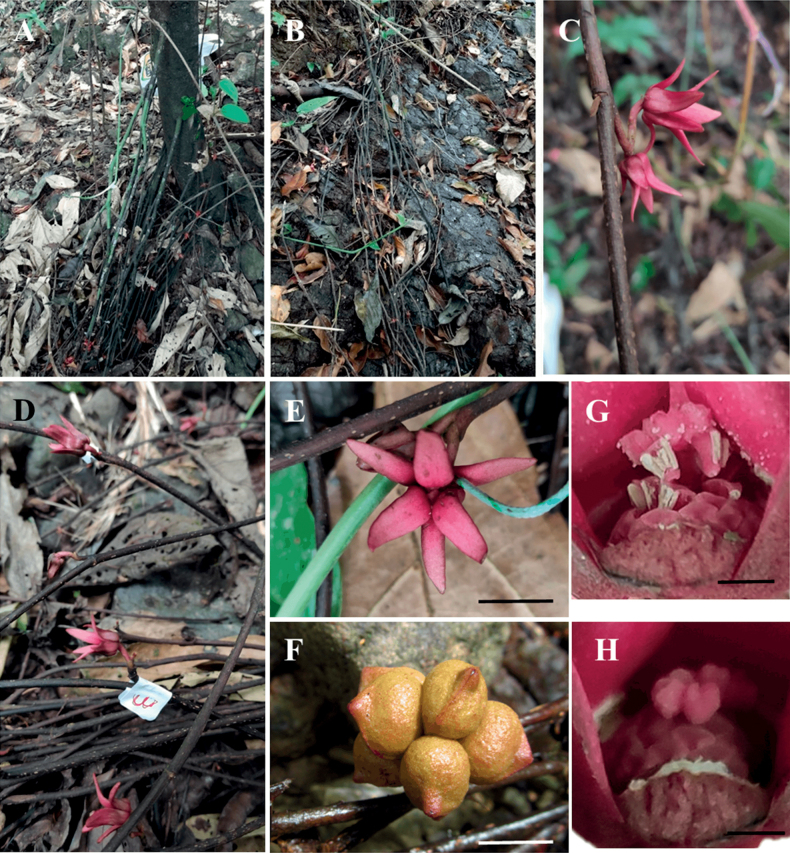
*Desmopsisterriflora* G.E.Schatz, T.Wendt, Ortiz-Rodr. & Martínez-Velarde **A** multiple flagella emerging from the base of the main trunk **B** flagella lying on the rocky ground **C, D** lateral inflorescences on the flagellum **E** flower in anthesis **F** monocarps **G** male flower releasing pollen **H** female flower.

Flagelliflory is a very rare phenomenon in nature and has been documented in ca. 20 tree species, most belonging to the Annonaceae and Moraceae families and restricted to the tropical forests of Asia, Africa, Mexico and South America (Schatz and Wendt 2004; Couvreur 2009; Harrison et al. 2012; Maas et al. 2015; Lobão 2017). Maas et al. (1993, 2003) distinguish two, non-homologous, types of flagelliform structures in Annonaceae: type 1: Flagella are specialized sympodial *branches* arising from the main trunk and characterized by the presence of poorly developed leaves, often reduced to bracts and by the successive lateral shoots ending in inflorescences; type 2: Flagella composed of internodal elongations of a single *inflorescence*, the extreme development of a sympodial rachis with all flowers growing towards the tips of the flagella. Very little is known about the origin and evolution of flagelliflory and about the ecological-evolutionary advantages that this condition provides to the species (Couvreur 2009). Some studies have shown that in species with flagelliflory, the flowers have a very low diversity of floral visitors and that pollinators and dispersers are specific species (Harrison et al. 2012; Teichert et al. 2012; Xicohténcatl-Lara et al. 2016).

In Mesoamerica (southern Mexico and Central America), flagelliflory has been documented in just one species of Annonaceae restricted to the karst forests of southern Mexico: *Stenanonaflagelliflora* T.Wendt & G.E.Schatz (Schatz and Wendt 2004). This species, which belongs to a clade of Central American genera within the larger south east Asian tribe Miliuseae (subfamily MalmeoideaeChatrou et al. 2012) is known from two allopatric localities in Veracruz, Mexico, one in the Uxpanapa-Chimalapas region and the other in the Los Tuxtlas region (Schatz and Wendt 2004; Xicohténcatl-Lara et al. 2016). *Stenanonaflagelliflora* is a small tree up to 2 m tall, with all its inflorescences borne on woody and branched flagella up to 3 m long, which often have small leaves or leaves reduced to bracts (Schatz and Wendt 2004). Its red flowers, with a reduced number of carpels, all with a single ovule, and its vegetative characteristics, clearly relate it to three clonal species of *Stenanona*, *S.humilis*, *S.monticola* and *S.wendtii*. The close relationship of these species was also corroborated by phylogenetic analyses of plastid data (Ortiz-Rodriguez et al. 2016).

Schatz (1987) mentions that a second species of Annonaceae with flagelliflory is present in Mexico. The species (a tree up to 8 meters tall) has a combination of characters such as distichous phyllotaxy, upper side of leaves with impressed to flat midrib, simple hairs indument, three or four sepals, pedicels basally articulated, a tiny bract at the base of the pedicels, bisexual flowers with red petals and fertile stamens, and a fruit consisting of indehiscent, two-seeded monocarps, that relate it to the Neotropical genera *Desmopsis*, *Stenanona* and *Sapranthus*. However, Schatz (1987) did not place the new species in either of those three genera (as they were delimited at that time), as the species showed a unique combination of characters that did conform to any of the genera (Table 1). Schatz (1987) tentatively named it *Uxpanapanonaflagellaris*, but this name was never formally described. This undescribed species is only known from two specimens collected more than 30 years ago in Veracruz, Mexico (collection number: *T. Wendt et al. 3125* and, *Schatz & Wendt 985*), so its taxonomic status at the genus level, its phylogenetic relationships and its conservation status remain unresolved.

**Table 1.** Comparison of diagnostic morphological characters of the unpublished name “*Uxpanapanonaflagellaris*” (Schatz 1987) and the genera of subtribe Sapranthinae.

Characters	“*Uxpanapanona*”	*Desmopsis*	*Sapranthus*	*Stenanona*
Number of petals	6, rarely 8	6	6	6, rarely 8
Number of sepals	3, rarely 4	3	3	3, rarely 4
Petals texture	Stiff and thick	Stiff and thick	Thin and fleshy	Thin and fleshy
Colour of petals	Red	Yellow, greenish or rarely white	Red to purple, rarely yellow or green	Red to purple, rarely yellow or white
Fused petals	No	No	No	No, rarely yes
Fused sepals	Yes, basely	Yes, basely	No	No, rarely yes
Food bodies	Present	Absent, rarely present	Present	Absent
Number of ovules per carpel	1 or 2	1 or 2, rarely several	Several	several, rarely 1 or 2
Leafy bracts	Absent	Present, rarely absent	Present, rarely absent	Absent
Monocarps shape	Globose	Globose	Cylindrical	Cylindrical
Wall of monocarps	Thick	Thin	Thick	Thick
Seeds ruminations	Spiniform	Peg-shaped	Lamelate	Spiniform
Pollen grains aperture	?	Disulcate	Disulcate	Inaperturate

Here, we carried out botanical explorations in Uxpanapa, Veracruz Mexico with the aim of finding individuals of this new species, documenting some aspects of its reproductive ecology, phylogenetic position and to describe it formally.

## ﻿Materials and methods

### ﻿Study area

**Figure 2.**
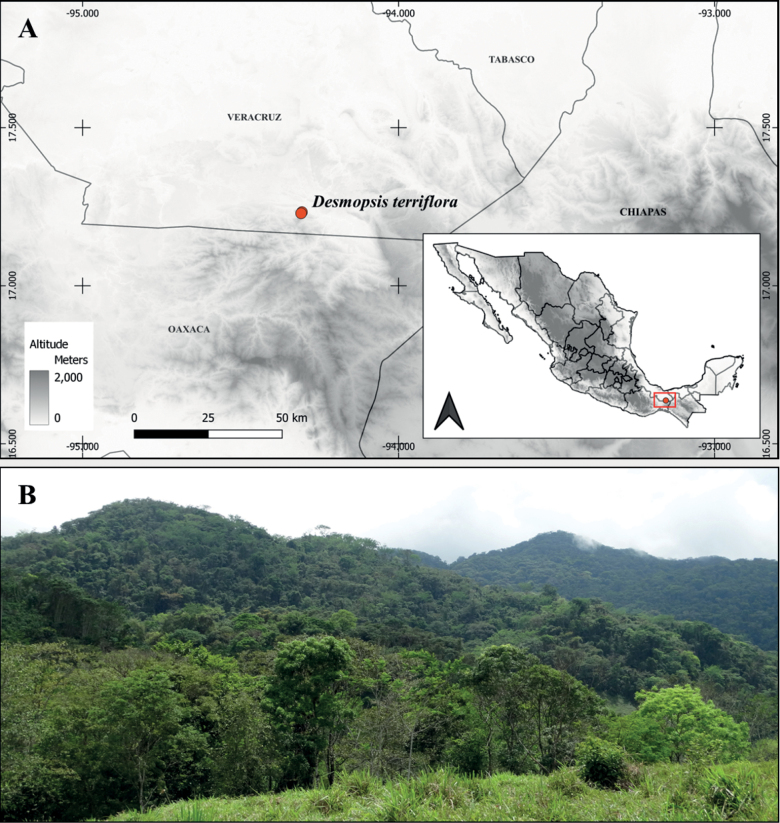
Geographic distribution of *Desmopsisterriflora* Schatz, T.Wendt, Ortiz-Rodr. & Martínez-Velarde **A** currently known localities for the species **B** tropical rainforest at Poblado 11 in Uxpanapa, Veracruz, México.

Field explorations to find the new flagelliflorous species were carried out in the region of Uxpanapa, Veracruz, Mexico (Fig. 2). The Uxpanapa region in southern Mexico between the states of Chiapas, Oaxaca and Veracruz, comprises species-rich, warm-humid forests that develop mainly on limestone karst soils (Wendt 1987, Fig. 2). These forests harbour a high number of endemic species and species that in Mexico are known only from the Uxpanapa region. The Uxpanapa region is considered one of the wettest areas in Mexico with mean annual precipitation greater than 4,000 mm (Wendt 1987). Unfortunately, the change in land use, forest exploitation, fragmentation and fires have had an impact on the connectivity and extension of the Uxpanapa forests and today they persist only in the form of isolated patches that are harder to access.

Based on the field notes of only one of the two collections, the species should occur around “El Poblado 11” (currently named Helio Garcia Alfaro) where it was collected in 1981 by Thom Wendt. Our first explorations around Poblado 11 were unsuccessful; the vegetation surrounding the town is very fragmented and consists of secondary vegetation. A community-led protected forest area in front of Poblado 11 offered a better chance of finding the species (Fig. 2). This protected area is part of the Ejido Progreso Chapultepec and is one of the few areas near Poblado 11 that still preserves original and fire-free vegetation. New explorations within this protected area of forest gave results and in 2014 we found a population of a large tree species with long flagelliflorous inflorescences, which we have been studying in detail since 2022. This species appeared to represent the undescribed taxon of Schatz (1987) relocated 30 years after its last collection.

### ﻿Phylogenetic position of the new species

The phylogenetic relationships of the new species were inferred using a hybrid-capture phylogenomic approach. Representatives of the genera *Desmopsis*, *Sapranthus*, *Stenanona* and *Tridimeris*, as well as some representatives of the Miliuseae tribe were included to assess the taxonomic status of this flagelliflorous species at the genus level (Suppl. material 2). As the phylogenetic hypotheses proposed so far suggest that *Stenanona* and *Desmopsis* are not monophyletic groups and their species appear intermingled in a strongly supported clade (Ortiz-Rodriguez et al. 2016), it is expected that if the new species is a separate genus as speculated by Schatz (1987), it will not cluster within the *Desmopsis*-*Stenanona* clade nor within the *Sapranthus* clade.

The Annonaceae baiting kit (Couvreur et al. 2019) was used for DNA sequencing. This kit targets a total of 469 Annonaceae wide exons. DNA extraction, DNA sequencing protocols, and the bioinformatic procedure are presented in Couvreur et al. (2019) and Breé et al. (2020). Thus, after filtering our data for the genes covering 75% of each exon for 75% of the individuals, and eliminating suspected paralogues following HybPier approach (see Couvreur et al. 2019; Breé et al. 2020), we ended up with a total of 323 genes used for the phylogenetic analyses.

Phylogenetic relationships among taxa were estimated using maximum likelihood (ML) methods performed with RAxML (Stamatakis 2014). We also ran an ASTRAL-III (v5.5.11) analysis (Zhang et al. 2017) as performed by Couvreur et al. (2019). The paired fastq sequences for individuals included in this study are available in Genbank SRA under Bioproject number PRJNA508895 (http://www.ncbi.nlm.nih.gov/bioproject/508895).

### ﻿Pollen characteristics

We analysed the pollen of the new species using scanning electron microscopy (SEM) at the Photography and Microscopy of the Biodiversity Laboratory 1, Universidad Nacional Autónoma de México (UNAM). The flowering material for SEM was collected and preserved in alcohol 70%. The material was dehydrated with gradual alcohol (ethanol) solutions at 70%, 80%, 96%, and 100%, 24 hours each. Then, the material was dried to the critical point using CO_2_, placed on aluminium sample holders, and covered with a layer of gold. Finally, specimens were observed under a scanning electron microscope (Hitachi- SU1510, Tokyo, Japan) at a voltage of 10 or 15 kw.

### ﻿Notes on reproductive ecology

Flower phenology was documented to determine the timing of the onset of stigmatic receptivity, its duration and the start and duration of the staminal phase (see Ratnayake et al. 2007). Also, extensive observations on mature flowers of different individuals were made. Observations were made every hour from 8:00 a.m. to 6:00 p.m. on 10 different individuals. The identity of the visitor, visitor behavior and stage of sexual maturity of the flower (pistillate: colour change and stigma exudate; staminate: dehiscence in anthers or stigmas fall) were recorded. External visitors and visitors inside the flower were captured when possible. To determine potential pollinators, we used a poisson regression modelling approach for handling the visit frequency data (Cameron and Trivedi 1998). We used generalized linear models (glm) using the frequency of visits per hour as response variables and each visitor as a factor [glm (variable ~species, family = “poisson”)]. For each analysis, we performed an Analysis of Deviance to determine differences within species (chi-square test, Fox and Weisberg 2019). Then, we carried out a Least-squares means test to assess differences between pairs of species (tukey p-adjust), using the “*emmeans*” function (Lenth 2022). A higher frequency of visits and presence of pollen grains of the new species on the visitors’ bodies was considered as evidence of potential flower pollinators.

## ﻿Results

### ﻿Phylogenetic relationships of the new species

**Figure 3.**
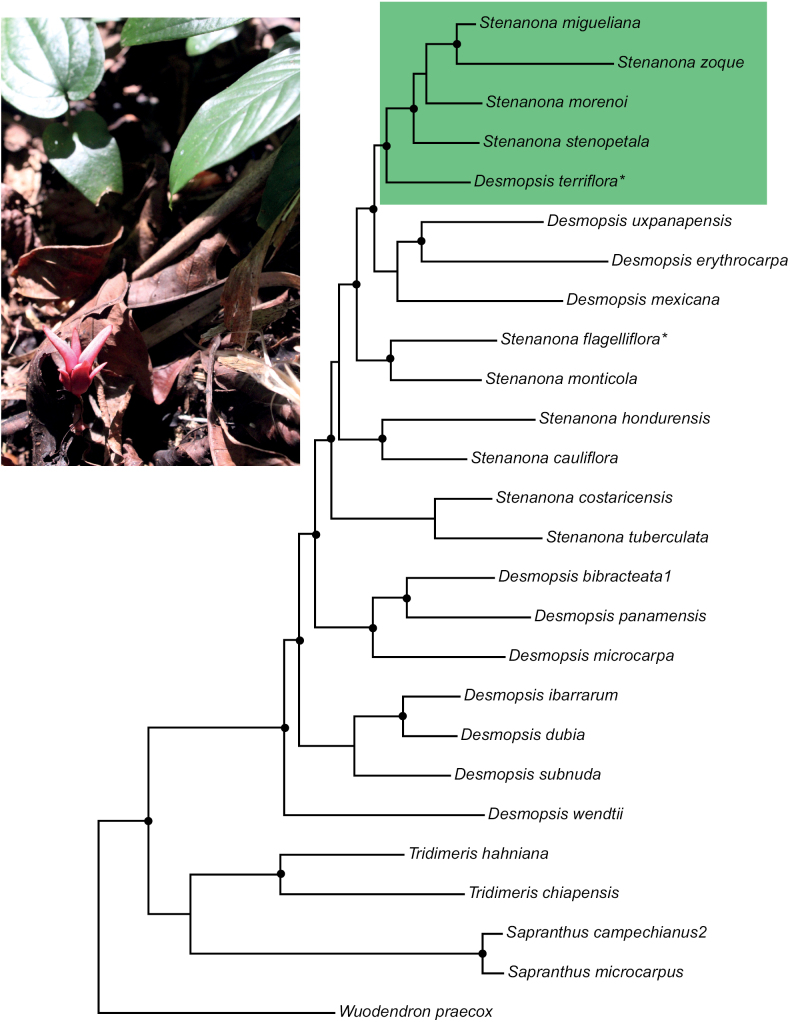
Maximum likelihood tree (RaxML) of the Neotropical clade of Miliuseae (subtribe Sapranthinae) based on 323 Annonaceae wide exons and with emphasis on the species of *Stenanona* Standl. and *Desmopsis* Saff. Darker circles indicate strongly supported clades (bootstrap values greater than 90 and Posterior Probabilities greater than 9.0). The shaded clade includes the new species.*= flagelliflorous species. In the photo, a flower of *Desmopsisterriflora* emerging from the ground is shown.

The ASTRAL and the ML (Fig. 3) analyses resulted in similar tree topologies. Our phylogenetic analysis recovered two strongly supported clades of Central American genera: the *Desmopsis*-*Stenanona* clade (bootstrap support, BS 100; Posterior Probabilities, PP 1.0) and the *Sapranthus*-*Tridimeris* clade (BS 100, PP 1.0). The genera *Desmopsis*-*Stenanona* are in fact recovered here as non-monophyletic. In the *Sapranthus*-*Tridimeris* clade, both genera were recovered as reciprocally monophyletic with maximum support (*Sapranthus*, BS = 100, PP 1.0; *Tridimeris*, BS = 100, PP 1.0). The phylogenetic hypothesis showed that the new species is not a new genus but clustered within the *Desmopsis*-*Stenanona* clade (Fig. 3). It is placed within a sub-clade of Mexican trees that includes species of *Stenanona* with long, awned petals (BS = 100, PP 1.0).

### ﻿Pollen characteristics

**Figure 4.**
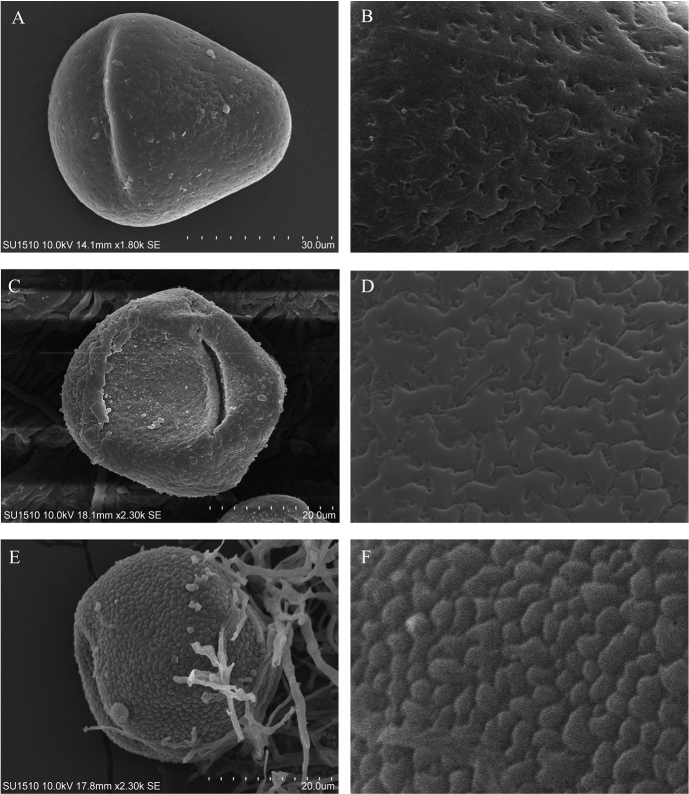
Scanning electron microscopy (SEM) images of pollen grains **A***Desmopsisterriflora* G.E.Schatz, T.Wendt, Ortiz-Rodr. & Martínez-Velarde, pollen grain without externally visible aperture(s) **B** exine sculpturing weakly rugulate to fossulate (-perforate) ornamentation **C***Desmopsistrunciflora* (Schltdl. & Cham.) G.E.Schatz, pollen grain showing one (another one at the opposite side likely to be also present) aperture exine area **D** exine sculpturing weakly rugulate to fossulate (-perforate) ornamentation **E***Stenanonatuberculata* G.E. Schatz & Maas, pollen grain without externally visible aperture(s) **F** exine sculpturing verrucate ornamentation.

Pollen grains of the new species are solitary, symmetrical, and ellipsoid, with two depressed germinative zones and are inaperturate. The grains are 40–45 μm long, 30–40 μm wide, with a weakly rugulate to fossulate (-perforate) exine ornamentation (Fig. 4).

### ﻿Notes on reproductive ecology

**Figure 5.**
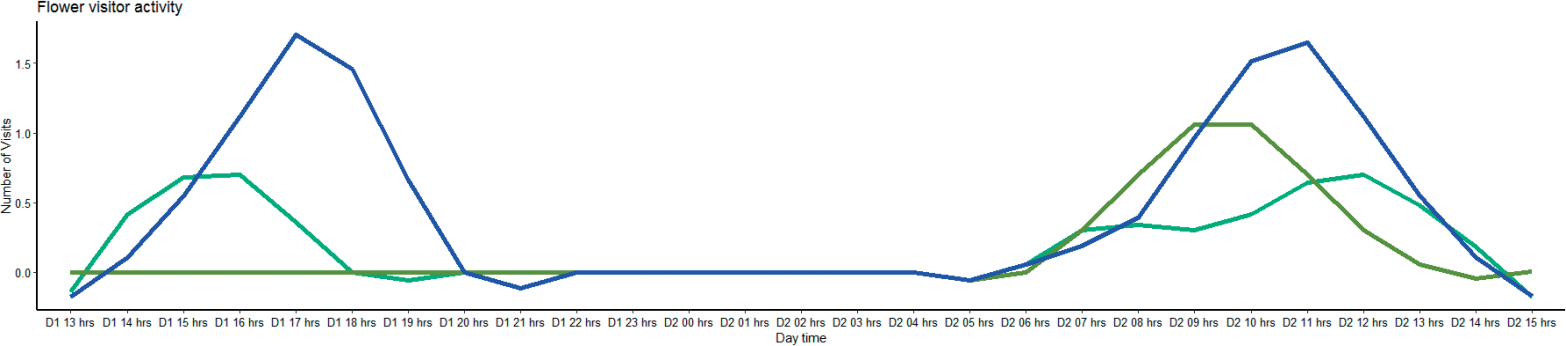
Timing of phenological events and visitor activity during sexually functional phases of *Desmopsisterriflora* flowers. The pistillate phase occurs between 1400 hours and 1800–1900 hours of the day, during this phase the floral visitors (light green: flies, blue: ants) are attracted by the strong banana-like odor in the flowers. The pistillate phase is succeeded by a long interim period of 11–12 h, during which the flowers were not sexually functional, neither floral scent dissipated, nor floral visitors observed. The staminate phase occurs between 1200–1300 hours of the second day, during which there is an obvious increase in floral scent and visitors are again attracted. Note that the beetles (dark green) are only present in the staminate phase.

The individuals of the new species were in full bloom during the observation period (April). All the individuals with flagella had inflorescences at different stages of development (36 ± 12.69 inflorescences per flagellum in 10 measured individuals). The number of flagella (7.7± 4.9 flagella per trunk) is positively correlated with the diameter of the main trunk (r^2^ = 51, p < 0.05). The length of the flagella (8 ± 4.0 m) is not directly associated with the diameter of the main trunk (r^2^ = 33, not significant), while the number of inflorescences is positively correlated to the length of the flagellum (r^2^ = 49, p < 0.05). The flowers were markedly protogynous, with the entire extent of reproductive activity restricted to a 27 hour-period. The pistillate phase lasted for 4–5 h. from ca. 1400 hours to 1800–1900 hours of a first day (Fig. 5). During this phase, the red inner petals closed so they formed a pollination chamber over the reproductive organs, while outer petals are extended or slightly reflexed (Fig. 1E), the stigmas seem enlarged, although they are not observed wet, nor with exudate; this phase was clearly correlated with the emission of a strong banana-like odour and with a further increase in floral visitors. The pistillate phase was succeeded by a long interim period of 11–12 h, during which the flowers were not sexually functional, neither floral scent dissipated, nor floral visitors observed (Fig. 5). The inner petals are more distant in this phase. The interim period was followed by a staminate phase (from the 0700 hours to the 1200–1300 hours of the second day) during which the petals closed once again to form a pollination chamber, the anthers dehisced, and there was an obvious increase in floral scent (Fig. 1G). The staminate phase ends with the detachment of the petals and the fall of all the stamens.

**Figure 6.**
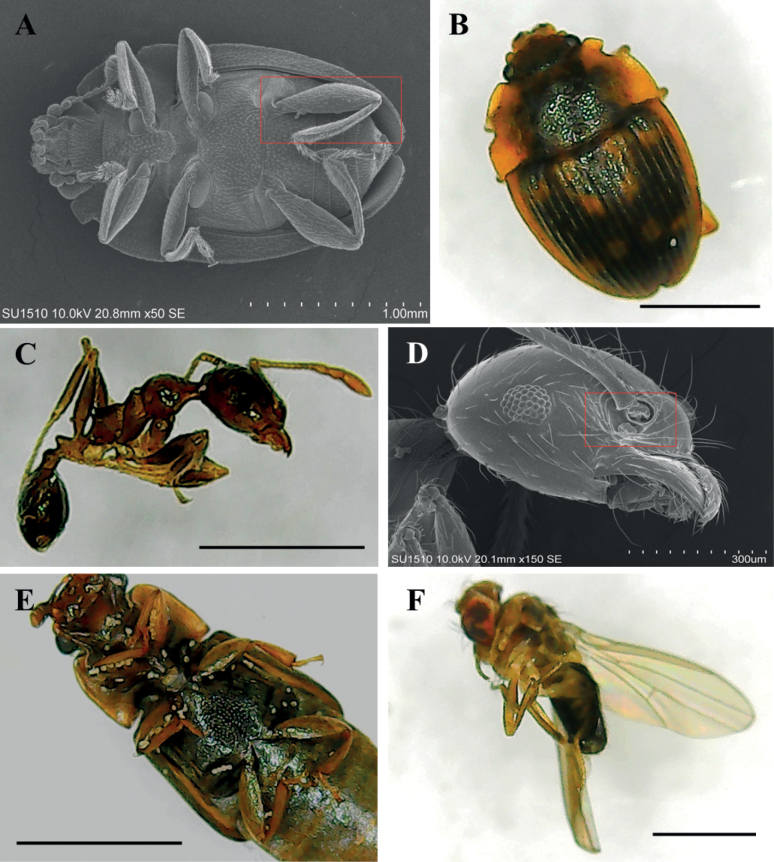
Scanning electron microscopy (SEM) pictures and stereoscopic images of the potential pollinators of *Desmopsisterriflora* flowers **A, B** unidentified beetles **C, D** unidentified ants **E** unidentified beetle **F** unidentified fly.

Throughout flower anthesis, low diversity and abundance of floral visitors were observed (9 insects’ observations in 27 hrs, 1 to 4 insects per observation). Floral visitors were insects from three main groups, flies, beetles, and ants. Flies were observed most often (7/9), followed by ants (5/9) and beetles (4/9) (Suppl. material 1). However, non-significant differences (tukey pairwise comparisons, P > 0.05) in number of visits were found between the three groups of insects. Both ants and beetles had pollen grains of the new species on their bodies (Fig. 6), but only the flies and ants were observed during the two flower phases (Fig. 5). Accordingly, flies and ants can be considered here as the most effective potential pollinator of the new species.

## ﻿Discussion

### ﻿Neither *Sapranthus* nor *Stenanona* – a new species of *Desmopsis*

The new species described here was suggested more than 35 years ago (Schatz 1987) to be part of a new genus. The combination of basely fused sepals, rigid petals with food bodies (abrupt thickening) at the base of the inner ones and its globose fruits with woody testa, prevented its inclusion in *Desmopsis*, *Sapranthus* or *Stenanona*, to which it is morphologically related (Schatz 1987, Table 1). After 35 years, knowledge about diversity, phylogenetic relationships and morphology of the genera involved has advanced considerably (Schatz and Maas 2010; Ortiz-Rodriguez et al. 2016; Schatz et al. 2018a, b; Ortiz-Rodriguez 2022). This currently allows the enigma of its phylogenetic position and taxonomic status to be resolved. The phylogenomic results presented here showed that the species is placed within the *Desmopsis*-*Stenanona* clade (Fig. 3), a lineage composed of *Desmopsis* and *Stenanona* species in which neither genera are monophyletic. Our phylogenetic results suggest that *Desmopsis* is the correct name for all species within this lineage (Schatz et al. in prep) and therefore we named the new species, *Desmopsisterriflora*. This decision is further supported by the pollen characteristics and floral morphology of *Desmopsisterriflora* (Fig. 4). The inaperturate pollen grains of the new species are a feature shared with all species of *Desmopsis* with red flowers (=*Stenanona*). However, in most of them, the exine sculpturing ornamentation is strongly rugulate-verrucate (Fig. 4). Thus, exine sculpturing ornamentation in the pollen grains of the new species is similar to that present in all species of *Desmopsis* with yellow-green flowers (most with aperturate grains) and in some species of *Stenanona* with pink or yellow flowers (*S.migueliana* and *S.zoque*) to which the new species is related (Fig. 4). Moreover, its flowers with stiff and thick (rigid) petals and the reduced number of ovules per carpel, are features also shared with most species of *Desmopsis* (Schatz et al. 2018a). Finally, some characteristics that prevented its inclusion in *Desmopsis*, such as the presence of an abrupt thickening in the base of the inner petals (food bodies) and the partial fusion of the sepals (Table 1), are present in the flowers of two species of *Desmopsis* endemic to Mexico (Ortiz-Rodriguez 2022).

### ﻿Flagelliflory in Mexico

Flagelliflory is rare phenomenon in nature and its presence in different lineages of angiosperms suggests an independent origin (Schatz and Wendt 2004). Even within the same genus or family, flagelliflory can occur due to convergence (Saunders 2010). Within the *Desmopsis*-*Stenanona* clade flagelliflory is now documented to occur in two species, *Stenanonaflagelliflora* and *Desmopsisterriflora* (Fig. 3). As suggested by the morphological characteristics, both species have the same type of flagellum (type 1, *sensu*Maas et al. 2003), specialized whip-like branches that trail along the ground or below it. Also both species share the red colour of the petals, and the reduction in the number of carpels and ovules (Table 2), features also present in other Annonaceae species with flagelliflory like *Duguetiaflagellaris* Huber, *D.sessilis* (Vell.) Maas (Maas et al. 2003) or *Isolonacauliflora* Verdc. (Couvreur 2009). Based on our phylogenetic results, the origin of flagelliflory within the *Desmopsis*-*Stenanona* clade is an independent phenomenon since *Stenanonaflagelliflora* and *Desmopsisterriflora*, are not sister species (Fig. 3). However, flagelliflory in both species could be linked to similar ecological constraints, Specifically, related to vegetative reproduction. It has been observed in the field that *Desmopsis*/*Stenanona* species can reproduce vegetatively when branches or secondary trunks detach from the main individual (observed in *S.migueliana* and *S.zoque*, species closely related to *Desmopsisterriflora*). Other species such as *Stenanonamonticola* (sister species of *S.flagelliflora*) usually clone by stolons. Thus, this is a line that deserves further investigation and could shed light on the origin and evolution of the flagelliflory.

**Table 2.** Morphological features of *Desmopsisterriflora* G.E.Schatz, T.Wendt,Ortiz-Rodr. & Martínez-Velarde compared to those of *Stenanonaflagelliflora* T.Wendt & G.E.Schatz.

Features	*Stenanonaflagelliflora*	*Desmopsisterriflora*
Size	Up to 2 m tall	up to 10 m tall
Leaves length	Up to 18 cm	up to 31 cm
Sepals	Free	Fused
Outer petals size	13–14 mm long	13–16 mm long
Pedicels	4–7 mm long	8–30 mm long
Food bodies	Absent	Present
Number of carpels	Up to 6	6–9
Ovules per carpel	1	1–2
Flagella length	Up to 3 m long	Up to 15 m long
Monocarps	Ellipsoid, thin wall	Globose, thick wall

### ﻿Does the flagelliflory mean pollinator specialization?

Our results on the reproductive ecology of *Desmopsisterriflora* suggest that flowers on flagella present a depressed visitation rate (low frequency and diversity of floral visitor), probably linked to the fact that the species has a very narrow distribution and is rare in the locality studied (Benadi and Pauw 2018). In addition, its flowers are mainly visited by flies, which could represent a certain degree of specialization considering that most Annonaceae species are visited only by beetles (Gottsberger 2012). Also, Xicohténcatl-Lara et al. (2016) reported flies as the main floral visitors of *Stenanonaflagelliflora*. Interestingly, an ecological study focused on *Duguetiacadaverica* (Annonaceae), another species with flagelliflory, supports a specialization hypothesis (Teichert et al. 2012). In that study, flowers of *D.cadaverica* are visited only by one species of small beetles. However, our results should be used with caution, flies as floral pollinators have been hypothesized for all the species of *Desmopsis* with red flowers (= *Stenanona*) (Schatz 1987) so that the higher frequency of flies in *Desmopsisterriflora* and *Stenanonaflagelliflora* flowers is not necessarily associated with the flagelliflory but an evolutionary convergence. Moreover, non-significant differences (tukey pairwise comparisons, P > 0.05) in number of visits were found between floral visitor of *Desmopsisterriflora*. Therefore, more studies are necessary to investigate this further.

### ﻿Taxonomic treatment

#### ﻿ Desmopsis terriflora

Taxon classificationPlantaeMagnolialesAnnonaceae

G.E.Schatz, T.Wendt, Ortiz-Rodr. & Martínez-Velarde sp. nov.

6FE0FD82-C36A-5FA3-A28D-1CA3EAF3DB1C

urn:lsid:ipni.org:names:77321861-1

Figs 1 , 2 , 3 , 4 , 5 , 6

##### Type.

Mexico. Veracruz, Municipio Uxpanapa, Ejido Progreso Chapultepec, 17°13'58.9"N, 94°18'20.5"W, 105 m, 26 Abril, 2022 (fl,yfr), *Ma. F. M. Velarde 72* (holotype MEXU; isotypes: MO, P).

##### Diagnosis.

*Desmopsisterriflora* is similar to *Stenanonaflagelliflora* since both species have flowers and inflorescences growing exclusively on flagella. *Desmopsisterriflora* differs from it by the combination of larger-sized individuals, flowers with rigid petals, food bodies at the base of the inner petals, fused sepals, a greater number of carpels and ovules per carpel, by its monocarps with a hard and woody testa, and its flagella up to 15 meters in length (Table 2).

***Trees***, 7–10 m tall, 12–45 cm DBH, bark dark green, verrucose *in vivo*, dark brown when dry, young branches and terminal shoots golden sericeous. ***Leaves*** membranaceous, alternate, distichous, broadly elliptic to oblong-elliptic, 17–31 cm long,4–10 cm wide, base obtuse to rounded, apex acute to acuminate, young leaves golden sericeous on both sides, mature leaves glabrous above and below, venation brochidodromus, 13–18 secondary veins per side, barely elevated to impressed above, raised below, the midrib impressed above but slightly canaliculate toward the base (sometimes with erect to appressed pale brown hairs), raised below and sparsely covered by golden brown hairs, petiole swollen, canaliculate, 5–8 mm long, sparsely covered by golden brown hairs. ***Inflorescences*** flagelliflorous, 5–20 woody flagella per trunk, glabrous, shoots emerging from the main trunk from near the base to 2 to 3 m high, then buried in the leaf litter just below the surface (subterranean), to 15 m long, branching; leaves and roots absent, apical parts of flagella greenish and thin, becoming progressively dark and woody toward the base, bearing perennial rhipidia at leafless nodes: the rhipidia terminal but lateral at each node because the flagellum keeps growing by a renewal shoot. Flower bearing part of the generally inflorescence erect, protruding above the leaf litter; ***Flowers*** 1 or 2 per inflorescence, flowering pedicels 8–30 mm long, densely covered by golden brown hairs, borne in the axil of a minute, clasping, ovate bract, and bearing a second minute, clasping ovate bract ca. midway, each bract 2 mm long, 2 mm wide, apex acute, outer surface densely covered by golden brown hairs, sepals 3 (rarely 4), valvate, connate at the base, triangular, 3–10 mm long,3–4 mm wide, apex obtuse, outer surface sparsely covered by minute golden brown hairs, glabrous on the inner surface, petals 6 (rarely 8), free, subequal, in two whorls, thick and stiff, pinkish-red, outer petals oblong-lanceolate 13–16 mm long,4–5 mm wide, apex obtuse to acute, outer surface sparsely covered by minute golden brown hairs, essentially glabrous on inner surface, margin ciliate, inner petals oblong-lanceolate, 17–20 mm long, 4–6 mm wide, apex acute, outer surface sparsely covered by minute golden brown hairs, essentially glabrous on inner surface, food bodies (an abrupt thickening) in the basal adaxial side, often with white striations below the food bodies, stamens ca. 30, 1.5–1.7 mm long, the filament 0.3 mm long, thecae 1–1.3 mm long, the anther connective 0.1 mm thick, either expanded discoid or prolonged into a deltoid ligulate appendage bent toward the gynoecium, carpels 6–9, stigmata globose, capitate to napiform, ca. 1 mm in diameter, covered by minute golden brown hairs, ovaries 1.2–1.6 mm long, densely covered by minute golden brown hairs, 1–2 ovules per carpel, lateral. ***Fruits*** apocarpous, consisting of 4–8, subsessile, globose monocarps, 1–2 cm in diameter, apex apiculate, base rounded, the longitudinal suture visible (ribbed), stipe up to 1 mm long, exocarp dark reddish to brown, ligneous when mature, endocarp yellow, coconut-scented at maturity. ***Seeds*** 1-(2) per monocarp, ellipsoid or rarely discoid, surface slightly rugose, endosperm ruminate (spiniform)1–1.5 cm long,1–1.7 cm wide.

##### Distribution.

*Desmopsisterriflora* is only known to date from the Uxpanapa region (Veracruz state) in southern Mexico (Fig. 2).

##### Habitat and ecology.

The species occurs in lowland tropical rainforests (100–200 m elevation) on karstic rock formations and on shallow soils, mainly occurring along ravines and river banks, protected from the wind and sun. It forms part of the middle stratum along with species of *Amphitecna* (Bignoniaceae), *Dialium* (Fabaceae), *Mortoniodendron* (Malvaceae) and *Terminalia* (Combretaceae). At the type locality, most individuals were found along a shallow river, and the flagella were all directed downstream and the woody fruits usually float on the water.

##### Phenology.

Flowering from April to May; fruiting between May and September.

##### Etymology.

The specific epithet “terriflora” refers to its flowers emerging from the ground.

##### Phylogenetic relationships.

*Desmopsisterriflora* is phylogenetically related to *Stenanonamigueliana*, *S.morenoi*, *S.stenopetala*, and *S.zoque* (Fig. 3), from which the new species is distinguished by its glabrous leaves (*versus* sparsely to densely pubescent), flagelliflorous habit (*versus* trunciflorous), the red color of its flowers (*versus* pink, yellow and white), the basal fusion of its sepals (*versus* totally free sepals), and by the reduced number of ovules (1 to 2) per carpel (*versus* several (5 to 10) ovules per carpel) (Ortiz-Rodriguez et al. 2018; Moreno-Méndez and Ortiz-Rodriguez 2020).

##### Additional specimens examined.

Mexico, Veracruz, Municipio Minatitlán, Lomas al S. de Poblado 11, ca. 27 km al E de La Laguna, 17°13'45"N, 94°18'30"W, 370 m, 1 April 1981 (fl), T. Wendt, A. Villalobos C. and I. Navarrete 3125 (CHAPA). loc. cit., 14 April 1984 (fl, yng fr), Schatz & Wendt 985 (MO); type locality, abril 2014 (fl, fr), Andres E. Ortiz-Rodriguez 783, 784 (MEXU).

## Supplementary Material

XML Treatment for Desmopsis terriflora

## ﻿Additional information

### Author ORCIDs

María Fernanda Martínez-Velarde https://orcid.org/0000-0001-9022-9658

Carlos Rodrigues-Vaz https://orcid.org/0000-0002-4263-3573

Vincent Soulé https://orcid.org/0009-0001-3910-2317

Francis J. Nge https://orcid.org/0000-0002-0361-8709

Thomas L. P. Couvreur https://orcid.org/0000-0002-8509-6587

Andrés Ernesto Ortiz-Rodriguez https://orcid.org/0000-0003-4583-6701

### Data availability

All of the data that support the findings of this study are available in the main text or Supplementary Information.

## Supplementary materials

Supplementary material 1 Total number of flower visits per hour Data type image (PDF file) Explanation note The fitted number of visits (in original scale) according to the results of the GLM analysis. https://binary.pensoft.net/file/867996This dataset is made available under the Open Database License (http://opendatacommons.org/licenses/odbl/1.0/). The Open Database License (ODbL) is a license agreement intended to allow users to freely share, modify, and use this Dataset while maintaining this same freedom for others, provided that the original source and author(s) are credited.María Fernanda Martínez-Velarde, Carlos Rodrigues-Vaz, Vincent Soulé, Francis J. Nge, George E. Schatz, Thomas L. P. Couvreur, Andrés Ernesto Ortiz-Rodriguez

Supplementary material 2 Country of origin and voucher information of the species used in the phylogenetic analyses Data type table (word document) https://binary.pensoft.net/file/867997This dataset is made available under the Open Database License (http://opendatacommons.org/licenses/odbl/1.0/). The Open Database License (ODbL) is a license agreement intended to allow users to freely share, modify, and use this Dataset while maintaining this same freedom for others, provided that the original source and author(s) are credited.María Fernanda Martínez-Velarde, Carlos Rodrigues-Vaz, Vincent Soulé, Francis J. Nge, George E. Schatz, Thomas L. P. Couvreur, Andrés Ernesto Ortiz-Rodriguez
