# Supplementary figures and images for: ﻿Desmopsisterriflora, an extraordinary new species of Annonaceae with flagelliflory
Source: PhytoKeys. 2023 Jun 23;227:181–98. doi: 10.3897/phytokeys.227.102279 (PMC10314296; doi:10.3897/phytokeys.227.102279)

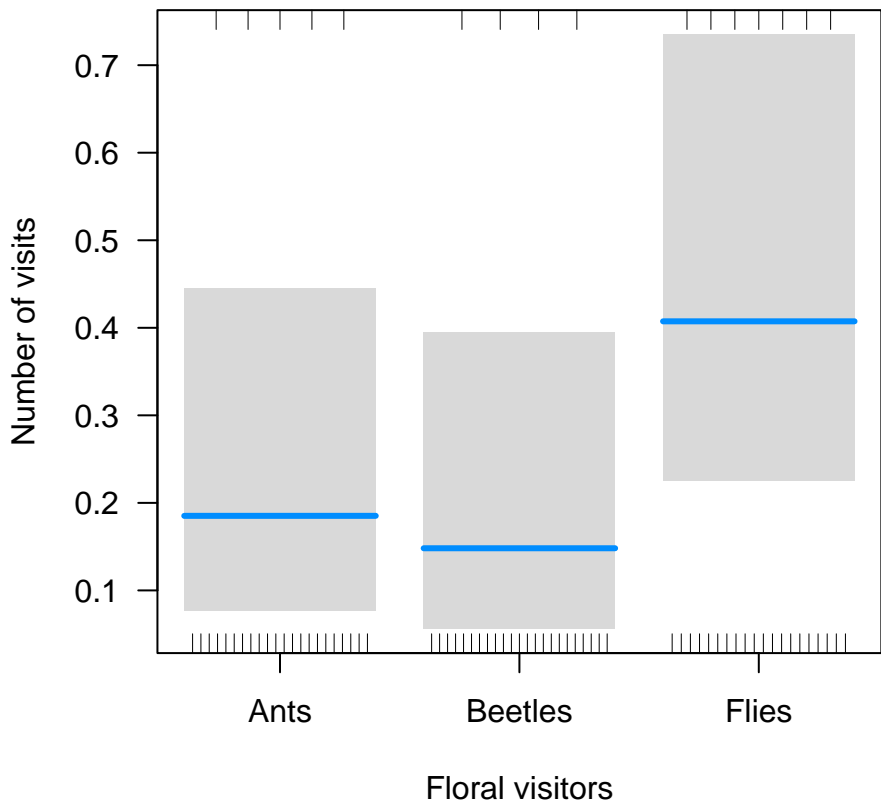

Supplement: Supplementary material 1 — Total number of flower visits per hour [file phytokeys-227-181_article-102279__-s001.pdf]
