# Supplementary material for: ﻿Desmopsisterriflora, an extraordinary new species of Annonaceae with flagelliflory
Source: PhytoKeys. 2023 Jun 23;227:181–98. doi: 10.3897/phytokeys.227.102279 (PMC10314296; doi:10.3897/phytokeys.227.102279)
Supplement: Supplementary material 2 — Country of origin and voucher information of the species used in the phylogenetic analyses [file phytokeys-227-181_article-102279__-s002.docx]

**Table S1.** Country of origin and voucher information of the species used in the phylogenetic analyses

| **Species** | **Country** | **Voucher (Herbaria)** |
| --- | --- | --- |
| *Phaeanthus splendens* Miq. | Malaysia | R.E. Onstein 73 (P) |
| *Meiogyne virgata* (Blume) Miq. | Malaysia | R.E. Onstein 43 (P) |
| *Miliusa umpangensis* Chaowasku & Kessler | Thailand | T. Chaowasku 89 (CMB) |
| *Alphonsea kinabaluensis* J. Sinclair | Malaysia | T.L.P. Couvreur 820 (P) |
| *Mitrephora maingayi* Hook.f. & Thomson | Indonesia | WJJO de Wilde 20152 (P) |
| *Stelechocarpus burahol* (Blume) Hook.f. & Thomson | Singapore | D.C. Thomas 3446 (SING) |
| *Sageraea lanceolata* Miq. | Malaysia | R.E. Onstein 63 (P) |
| *Polyalthia* *cauliflora* Hook.f. & Thomson | Singapore | D.C. Thomas 3463 (SING) |
| *Popowia alata* S.K. Ganesan | Malaysia | H. Sauquet 180 (P) |
| *Wuodendron praecox* (Hook.f. & Thomson) B.Xue, Y.H.Tan & X.L.Hou | Thailand | T. Chaowasku 108 (CMUB) |
| *Tridimeris hahniana* Baill. | Mexico | G.E. Schatz, 1198 (MO) |
| *Tridimeris chiapensis* M.A.Escobar & Ortiz-Rodr. | Mexico | H. Gómez, 3862 (MEXU) |
| *Sapranthus microcarpus* (Donn. Sm.) R.E.Fr. | Mexico | A.E. Ortiz-Rodríguez, NA (MEXU) |
| *Sapranthus campechianus* (Kunth) Standl. | Mexico | A.E. Ortiz-Rodríguez 1321 (MEXU) |
| *Desmopsis wendtii* G.E.Schatz | Mexico | G.E. Schatz, 988 (MO) |
| *Desmopsis subnuda* (R.E.Fr.) G.E.Schatz & Maas | Mexico | A.E. Ortiz-Rodríguez, 767 (MEXU) |
| *Desmopsis ibarrarum* G.E. Schatz ex Ortiz-Rodr. | Mexico | A.E. Ortiz-Rodríguez 765 (MEXU) |
| *Desmopsis dubium* Ortiz-Rodr. & Espinosa-Jim. | Mexico | A.E. Ortiz-Rodríguez, 785 (MEXU) |
| *Desmopsis microcarpa* R.E.Fr. | Colombia | P.J.M. Maas, 10483 (WAG) |
| *Desmopsis bibracteata* (B.L. Rob.) Saff. | Costa Rica | R. Liesner 15117 (U) |
| *Desmopsis panamensis* (B.L.Rob.) Saff. | Costa Rica | ML, 3 (MEXU) |
| *Stenanona costaricensis* R.E.Fr. | Costa Rica | L.W. Chatrou 67 (U) |
| *Stenanona tuberculata* G.E.Schatz & Maas | Mexico | A.E. Ortiz-Rodriguez 742 (MEXU) |
| *Stenanona hondurensis* G.E.Schatz, F.G.Coe & Maas | Honduras | R. Aguilar 4062 (NY) |
| *Stenanona cauliflora* (J.W.Walker) G.E.Schatz | Mexico | A.E. Ortiz-Rodríguez, 787 (MEXU) |
| *Stenanona flagelliflora T.Wendt & G.E.Schatz* | Mexico | A.E. Ortiz-Rodriguez 789 (MEXU) |
| *Stenanona monticola* Maas & G.E.Schatz | Mexico | A.E. Ortiz-Rodríguez, 802 (MEXU) |
| *Desmopsis mexicana* R.E. Fr. | Mexico | G.B. Hinton 15836 (NY) |
| *Desmopsis uxpanapensis* G.E. Schatz | Mexico | A.E. Ortiz-Rodríguez 780 (MEXU) |
| *Desmopsis erythrocarpa* Lundell | Guatemala | E. Contreras 6809 (P) |
| *Desmopsis terriflora* | Mexico | A.E. Ortiz-Rodríguez, 783 (MEXU) |
| *Stenanona stenopetala* (Donn.Sm.) G.E.Schatz | Belize | G. Davidse, 35703 (MO) |
| *Stenanona morenoi* Ortiz-Rodr. & Mor.-Ménd. | Mexico | GMM 87 (MEXU) |
| *Stenanona migueliana* Ortiz-Rodr. & G.E.Schatz | Mexico | A.E. Ortiz-Rodríguez, 796 (MO) |
| *Stenanona zoque* Ortiz-Rodr. & H.Gómez | Mexico | H. Gómez, 3858 (MEXU) |
